# 

**Published:** 1849-07

**Authors:** 


*To Correspondents.*—The report of a very interesting case from Dr. A. C. Dayton of Vicksburg, Miss., was received too late for publication in the present number of the Journal. It shall appear in the next. In reply to the question of S. J.—"Can the gold foil of Messrs Abbey & Son be obtained in Baltimore?" we answer yes. Messrs Armstrong & Berry are their agents, and sell it at their prices. We decline publishing the communication of A. M. The parties immediately concerned are the only ones that would be at all interested in the matter to which it refers. To the question, as propounded by half a dozen or more correspondents: "What has become of the proceedings of the last meeting of the American Society of Dental Surgeons?" We answer, we have just received a letter from Dr. Westcott, saying, that he had recently received part of them from one of the members of the society, and had been waiting for the remainder, before sending them to us for publication. He, however, stated he would send what he had, if we thought it would do to publish a part without the whole, but the letter of Dr. W. was received too late, to obtain them in time for the present number.—*Balt. Ed.*

*To Agents and Subscribers.*—Such of our agents as have any of the Journal's money in their hands, are respectfully requested to remit the same to us, or to the treasurer of the American Society of Dental Surgeons, Dr. Dunning, of No. 1 Bond-st., New York. Delinquent subscribers are also requested to remit the amount of their arrears immediately on the receipt of this No.—*Balt. Ed.*

### INDEX.

PAGE.

A

Arching a Plug over an Exposed Nerve, 39
Acutenaculum, 67
Atmospheric Pressure for Confining Artificial Teeth, 77
Artificial Palates and Velums, 80
Antrum Maxillare, &c., Application of Lunar Caustic to, in Tic Douloureux, 86
Antrum Maxillare, Perforation of, 103
Antrum Highmorianum, Abscess of, &c., 106
Atrophine in Painful Affections of the Face, 133
American Society of Dental Surgeons, Ninth Meeting of, 143
Address to the Graduates of Balt. Col. of Dental Surgery, 223
Address to the Graduating Class of Balt. College of Dental Surgery, Valedictory, 229
Alumni of Balt. College of Dental Surgery, Constitution and By-Laws of the Society of, 248
Alumni of Balt. Col., Proceedings of First Meeting of, 261
Asbestos and Collodion in Stopping Teeth, 255, 388
Air-chamber Plates, Cleveland's, 263
Anæsthesia, 263
Associated Alumni of the Balt. College of Dental Surgery, 264
Address delivered before the Associated Alumni of the Balt. College of Dental Surgery, by J. H. Foster, M. D., 265
Anæsthesia, or the Employment of Chloroform and Ether in Surgery, &c., 303
American Society of Dental Surgeons, Tabular Sheet of the, 305
Amalgam for Filling Teeth, a New, 307, 373
Austen, P. H., M. D., Remarks on the Use of Clasps, &c., by, 357

PAGE.

Analysis, Chemical, Qualitative and Quantitative, by Henry M. Noad, 372
American Society of Dental Surgeons, Postponement of Tenth Meeting of the, 373
Anatomy, Specimens of Morbid Dental, 373
Addington on the Principles and Practice of Dentistry, 303
Artificial Teeth mounted on Plate, over Diseased Roots, 381
Agents and Subscribers, to, 383

B

Bistoury, Velum, 67
Brown, B. B., M. D., Observations on Hemorrhage, by, 108
Blow-Pipe, Lawrence's Improved Portable, 141, 256
Bond, T. E., M. D., Introductory Lecture by, 169
Baltimore College of Dental Surgery, Annual Commencement of the, 256
Baltimore College of Dental Surgery, Associated Alumni of the, 264
Baltimore College of Dental Surgery, 377

C

Cone, C. O., D. D. S., Report on Practical Dentistry, by, 3
Cheek, Foreign Body lodged in the, 134
Cancrum Oris, 135
Cures for Cholera, 138
Cholera, Progress of the, 139
Composition of Salivary Calculus taken from a Horse, 142
Cholera, 152
Capping Exposed Dental Nerve with Gold Plate, 36
Curved Forceps, 66
Collodion in Asbestos, Use of, in Stopping Teeth, 255, 388

PAGE.

Cleveland's air-chamber Plates, 262
Cleveland's Wax-holders, 263
Course of Lectures on Dental Physiology and Surgery, by John Tomes, 301
Compound Screw Forceps, Dr. Hullihen's, 306, 335
Casket and the Ribbon, 307, 309
Cone, C. O., M. D., on Treatment of Dental Caries when it extends nearly to the Pulp-Cavity, 349
Clasps, Remarks on the Use of, 357
Cook, A., Essay on the Teeth by, 371
Chemical Analysis, &c., 372
Chair, New Operating, 376
College of Dental Surgery, Baltimore, 377
Clark's Patent Method of Inserting Pivot Teeth, 377
Correspondents, To, 383
Carious Teeth, Strictures on the Destruction of the Nerves in the Treatment of, 386

D

Dentistry, Practical, Report on, by C. O. Cone, M. D., 3
Dentition, Third, 24
Decay in Teeth, by J. Lee, 96
Dwinelle, W. H., D. D. S., Perforation of the Antrum by, 103
Dental Surgeons of the State of New York, Proceedings of Society of, 124
Dental Surgeons, Report of the Proceedings of the Pennsylvania Society of 128
Diagnosis of Hypertrophy of the Brain from Chronic Hydrocephalus, 142
Dental Surgeons, Ninth Meeting of the Am. Society of, 143
Dental Surgeons, Fourth Annual Meeting of the Mississippi Valley Association of, 154
Dental Education, by Dr. Handy, 363
Dental Physiology and Surgery, by John Tomes, 301
Dictionary of Dental Science, Dr. C. A. Harris', 301

PAGE.

Dentistry, Principles and Practice of, Simplified and Condensed, by Dr. R. D. Addington. 303
Dental Operations, Ambler's Journal of, 308
Dwinelle, W. H., M.D., D.D.S. The Casket and the Ribbon, or the Honors of Ether, by, 309
Dubs' Patent Screw Forceps, 335
Dental Caries, Treatment of, by Dr. C. O. Cone, 349
Diseases of the Mouth, by Hattute, 372
Dental Anatomy, Morbid, Specimens of, 373
Dental Surgery, Baltimore College of, 377
Dentists, Impositions Practiced by, 380
Digestion, Influence of the Pneumo-gastric Nerves, on, 387

E

Exposed Dental Nerve, Plugging over, 36
Exposed Dental Nerve, Capping with a Gold Plate, 38
Exposed Dental Nerve, Arching a Plug over, 39
Exostoses, their Character, 133
Elliot's File Carrier, 152
Education, Dental, by Dr. Handy, 363
Elongation of the Under Jaw, and Distortion of the Face and Neck, by Dr. Hullihen, 157
Etherization, on the Progress of, 191
Employment of Chloroform and Ether in Surgery and Midwifery, 303
Evans' New Amalgam, Remarks on, 307, 373
Extract of a Letter from Mr. Andrew Wilson, 338
Exposed Pulps, Filling Teeth over, by Dr. Harris, 340
Erasmus Wilson, Portraits of the Skin, by, 370
Essay on the Teeth, by A. Cook, 371
Early Operations for Hare-lip and Cleft Palate, 72

PAGE.

F

Fang, Plugging to the End of, 43
Forceps, Curved, 66
Face, on the Action of Atrophine in Painful Affections of the, 133
Foreign Bodies lodged in the Cheek for ten months, 134
Face and Neck, Distortion of, 157
First Annual Meeting of the Society of the Alumni of the Balt. Col. of Dental Surgery, 261
Forceps, Dubs' Patent Screw, 335
Filling Teeth when the Pulp-Cavity is Exposed, 340
Filling Teeth, Mr. Evans' Amalgam for, 373
Filling Teeth, a New Amalgam for, 307

H

Hemorrhage, 61

Hare-Lip and Cleft Palate, Early Operations for, 72

Hill, Dr., Letter from, 82

Hemorrhage, Observations on, by B. B. Brown, 108

Hypertrophy of the Brain from Chronic Hydrocephalus, Diagnosis of, 142

Hullihen's Compound Screw Forceps, 306

Harris, C. A., M. D., on Filling Teeth over Exposed Pulps, 340

Handy, W. R., M. D., on Dental Education, 363

Horny Growth from the Head in the Human Subject. 388

I

Impressions, Taking of, 75

Introductory Lecture, delivered before the Class of the Medical Department of the Washington University, by T. E. Bond, A. M., M. D., 169

Inhalation of Ether in Operations, 370

Incisor, Malformation of a Lower, 376

Inserting Pivot Teeth, Mr. Clark's Patent Method of, 377

Immobility of the Orbicularis Oris, 378

PAGE.

Imposition Practiced by Dentists, 380
Improved Portable Blow-pipe, Lawrence's 141, 256
Indian Hemp in Facial Neuralgia, 387

J

Jaw, Upper, Cases of Affection of, 136
Journal, the Library Part of our, 151, 380
Journal, Agents of, 153
Jaw, Elongation of the Under, &c., 157
Journal of Dental Operations, Ambler's, 308

L

Letter from Dr. A. Hill, Norwalk, Ct., 82
Lunar Caustic, Cases of Tic Douloureux treated with, 86
Lee, Dr. J., on Decay of Teeth, 96
Lawrence's Improved Portable Blow-pipe, 141, 256
Library Part of our Journal, 151
Lectures on Dental Physiology and Surgery, by J. Tomes, 301
Letter from Mr. Andrew Wilson, 338
Lower Incisor, Malformation of, 376
Lymphatic Vessels of the Tongue, 384

M

Medical Lexicon of Modern Terminology, 303
Mineral Teeth, Manufacture and Mounting of, 165
Manufacturers, Notice to, 255
Morbid Dental Anatomy, Specimens of, 373

N

New Amalgam for Filling Teeth, 307
New Amalgam, Remarks on Mr. Evan's, 307
New Remedy for Tooth-ache, 228
Notice to Manufacturers, 255

PAGE.
Ninth Annual Meeting of the American Society of Dental Surgeons, 143
Notice upon some of the Diseases of the Mouth, &c. 372
New Operating Chair, 376
Nasmyth, Dr. Alexander, 380
Necrosis and Expulsion of the Os Hyoides, 135

O

Observations on Hemorrhage, 108
Obituary, 264, 383, 385
Osseous Union of Two Teeth, 307
Operating Chair, 376
Orbicularis Oris, Partial Immobility of, 378

P

Plugging the Teeth, 28
" over an Exposed Nerve 36
Plugging the Organ to the End of the Fang, 43
Palatine Deficiencies, Treatment of, 65
Pivoting an Artificial Crown on a Natural Fang, 73
Pivot Gauge, 74
Principles and Practice of Dentistry, 303
Pelican, 306
Progress of Etherization, 191
Portable Blow Pipe Lawrences, 141, 256
Points in the Treatment of Hare-lip, 253
Plates, Cleveland's Air Chamber, 262
Practical Dentistry, Report on, by C. O. Cone, 3
Perforation of the Antrum, 103
Progress of the Cholera, 139
Practical Hints, 147
Patent Screw Forceps, Dub's, 335
Portraits of the Skin, by Erasmus Wilson, 370
Postponement of the Meeting of American Society of Dental Surgeons, 373
Pivot Teeth, Dr. Clark's Patent Method of Inserting, 377
Partial Immobility of the Orbicularis Oris, 378

PAGE.
Professional Items, 382
Professional Piracy, a Charge of 382
Pneumogastric Nerves, Influence of the, on Digestion, 387

R

Report on Practical Dentistry, by Dr. C. O. Cone, 3
Report of the Proceedings of the Pennsylvania Society of Dental Surgeons, 128
Remedy for Tooth-ache, 228
Remarks on Mr. Evan's New Amalgam, 373

S

Skin, Portraits of, by Erasmus Wilson, 370
Some of the Diseases of the Mouth, 372
Specimens of Morbid Dental Anatomy, 373
Springing of Atmospheric Pressure Plates while Soldering, 378

T

Tic Douleureux, treated with Lunar Caustic, 86
Teeth, Decay in, 96
" Plugging the 28
" Extraction of, 52
Treatment of Palatine Deficiencies, 65
The Curved Forceps, 66
Taking Impressions, 75
Tooth-ache, a New Remedy for, 228
Treatment of Hare-lip, 253
Tabular Sheet of the American Society of Dental Surgeons, 305
Teeth, an Essay on, by A. Cook 371

V

Velum Bistoury, 67
Valedictory Address to the Graduating Class of the Balt. College of Dental Surgeons, by E. B. Gardette, M. D., 229

W

Wax-holders, Cleveland's, 263

